# HCV Genotypes Are Differently Prone to the Development of Resistance to Linear and Macrocyclic Protease Inhibitors

**DOI:** 10.1371/journal.pone.0039652

**Published:** 2012-07-06

**Authors:** Valeria Cento, Carmen Mirabelli, Romina Salpini, Salvatore Dimonte, Anna Artese, Giosuè Costa, Fabio Mercurio, Valentina Svicher, Lucia Parrotta, Ada Bertoli, Marco Ciotti, Daniele Di Paolo, Cesare Sarrecchia, Massimo Andreoni, Stefano Alcaro, Mario Angelico, Carlo Federico Perno, Francesca Ceccherini-Silberstein

**Affiliations:** 1 Department of Experimental Medicine and Biochemical Sciences, University of Tor Vergata, Rome, Italy; 2 Department of Pharmacobiological Sciences, University of Catanzaro “Magna Græcia”, Catanzaro, Italy; 3 Complex Unit of Molecular Virology, Tor Vergata University Hospital, Rome, Italy; 4 Department of Internal Medicine, Tor Vergata University Hospital, Rome, Italy; 5 Department of Public Health, Tor Vergata University Hospital, Rome, Italy; INSERM, France

## Abstract

**Background:**

Because of the extreme genetic variability of hepatitis C virus (HCV), we analyzed whether specific HCV-genotypes are differently prone to develop resistance to linear and macrocyclic protease-inhibitors (PIs).

**Methods:**

The study includes 1568 NS3-protease sequences, isolated from PI-naive patients infected with HCV-genotypes 1a (N = 621), 1b (N = 474), 2 (N = 72), 3 (N = 268), 4 (N = 54) 5 (N = 6), and 6 (N = 73). Genetic-barrier was calculated as the sum of nucleotide-transitions (score = 1) and/or nucleotide-transversions (score = 2.5) required for drug-resistance-mutations emergence. Forty-three mutations associated with PIs-resistance were analyzed (36A/M/L/G-41R-43S/V-54A/S/V-55A-Q80K/R/L/H/G-109K-138T-155K/Q/T/I/M/S/G/L-156T/V/G/S-158I-168A/H/T/V/E/I/G/N/Y-170A/T-175L). Structural analyses on NS3-protease and on putative RNA-models have been also performed.

**Results:**

Overall, NS3-protease was moderately conserved, with 85/181 (47.0%) amino-acids showing <1% variability. The catalytic-triad (H57-D81-S139) and 6/13 resistance-associated positions (Q41-F43-R109-R155-A156-V158) were fully conserved (variability <1%). Structural-analysis highlighted that most of the NS3-residues involved in drug-stabilization were highly conserved, while 7 PI-resistance residues, together with selected residues located in proximity of the PI-binding pocket, were highly variable among HCV-genotypes. Four resistance-mutations (80K/G-36L-175L) were found as natural polymorphisms in selected genotypes (80K present in 41.6% HCV-1a, 100% of HCV-5 and 20.6% HCV-6; 80G present in 94.4% HCV-2; 36L present in 100% HCV-3-5 and >94% HCV-2-4; 175L present in 100% HCV-1a-3-5 and >97% HCV-2-4). Furthermore, HCV-3 specifically showed non-conservative polymorphisms (R123T-D168Q) at two drug-interacting positions. Regardless of HCV-genotype, 13 PIs resistance-mutations were associated with low genetic-barrier, requiring only 1 nucleotide-substitution (41R-43S/V-54A-55A-80R-156V/T: score = 1; 54S-138T-156S/G-168E/H: score = 2.5). By contrast, by using HCV-1b as reference genotype, nucleotide-heterogeneity led to a lower genetic-barrier for the development of some drug-resistance-mutations in HCV-1a (36M-155G/I/K/M/S/T-170T), HCV-2 (36M-80K-155G/I/K/S/T-170T), HCV-3 (155G/I/K/M/S/T-170T), HCV-4-6 (155I/S/L), and HCV-5 (80G-155G/I/K/M/S/T).

**Conclusions:**

The high degree of HCV genetic variability makes HCV-genotypes, and even subtypes, differently prone to the development of PIs resistance-mutations. Overall, this can account for different responsiveness of HCV-genotypes to PIs, with important clinical implications in tailoring individualized and appropriate regimens.

## Introduction

Chronic hepatitis C virus (HCV) infection remains one of the most pressing health emergencies worldwide, with an estimated global prevalence of more than 170 million people [1]. Despite its devastating impact on cirrhosis and hepatocellular carcinoma, therapeutic options are still limited. Up to 2011, the standard of care treatment for HCV infection was represented by a combination therapy of peg-interferon and ribavirin [2]. Sustained virologic response (SVR) to this regimen was associated with improved liver histology, as well as clinical benefits and mortality [3], [4]. However, nearly 50–60% of treated patients infected with the most prevalent genotypes HCV-1a and HCV-1b failed to achieve SVR [4]–[7].

The consequent need for innovative therapeutic strategies, has led to the development of several specifically-targeted antiviral drugs, directed against essential HCV proteins [8]. Among these, two NS3-protease inhibitors (PIs), boceprevir and telaprevir, are now approved for clinical use [9] and several other PIs are in development or in clinical trials [10]. These firtst two PIs have been evaluated in early-phase clinical-trials alone and in combination with peg-interferon and ribavirin, appearing to be highly effective in SVR [11]–[17].

Nevertheless, these encouraging data have been tempered by studies demonstrating either a differential sensitivity of HCV genotypes to PI-based therapy and an early selection of resistant variants.

Several factors, such as the inadequate fidelity and lack of proof-reading activity of the RNA-polymerase, the high genetic variability of HCV (31%–33% nucleotide difference among the 6 known HCV-genotypes and 20%–25% among the nearly 100 HCV-subtypes), and its high replication rate (10^10^–10^12^ virions/day produced in an infected-patient), can indeed have the ability to affect the efficacy of anti-HCV treatment, compromising the achievement of a SVR and strongly increasing the risk of drug-resistance development [18]–[20].

The first PIs, have been developed on the basis of HCV-1 NS3-protease structure and indeed showed reduced efficacy in clinical trials including other HCV-genotypes. For instance, the first PI BILN-2061 was found to be substantially less effective in individuals infected with HCV-2-3 [21]–[23]. Telaprevir also showed potent activity against HCV-1, less efficacy against HCV-2, and almost no efficacy against HCV-3-4-5 genotypes *in vitro* and *in vivo* [10], [24]–[26]. Similarly, recent *in vitro* results showed marked differences in susceptibility of different genotypes also to macrocyclic inhibitors, such as danoprevir, vaniprevir and TMC435 [10], [24], [26]. On the contrary, within a small pilot study, boceprevir monotherapy (400 mg TID) recently resulted in a 1.37 and 1.7 log HCV-RNA reduction in HCV-2 and HCV-3 infected patients respectively, a decrease similar to that observed in HCV-1 subjects receiving the same monotherapy dose (M. Silva et al., presented at APASL 2011). Boceprevir also showed similar efficacy when tested *in vitro* against several isolates from HCV genotypes 2a, 3a, 5a, 6a, with less pronounced changes against HCV-3 than telaprevir or other macrocyclic PIs [26].

Differences were also observed at the level of HCV-subtypes. Indeed, during clinical trials, selection of resistant variants to first-generation PIs and viral breakthrough were observed consistently more frequently in patients infected with HCV-1a than HCV-1b [27]–[29], and drug-resistant-variants emerged at frequencies of 5 to 20% of the total virus population as early as the second day after the beginning of treatment when either boceprevir or telaprevir were used as monotherapy [30].

Fourteen positions have been previously reported as involved in the development of major and minor PI-drug resistance mutations to either linear (positions 36, 54, 55, 109, 158, 170), macrocyclic (positions 80, 138, 168) or both classes of PIs (positions 41, 43, 54, 155, 156) [8], [10], [20], [31]–[35].

While for HCV-1a and HCV-1b the different antiviral activity, viral-breakthrough and selection of resistant-variants to telaprevir, boceprevir or danoprevir (a new macrocyclic PI) have been associated with nucleotide-variability at position 155 (resulting in a lower genetic-barrier for the development of R155K resistance-mutation in HCV-1a) [10], [20], [36],[37], the reason of a lower efficacy of PIs in HCV-2-3-4 is still largely unknown.

Considering these data, it is indeed conceivable that the genetic variability among HCV genotypes would have a great importance in HCV sensitivity to PIs, determining drug efficacy and even a different rate of selection of pre-existing resistant HCV variants [10], [28], [38], [39]. However, the characterization of HCV genetic variability at NS3 positions critical for PIs drug-resistance is still missing, especially in non-1 HCV genotypes.

Therefore, the aim of this study was to define, at either nucleotide or amino acid level, the HCV-NS3 genetic variability, among all different HCV-genotypes and subtypes commonly spread worldwide, focusing attention on codons associated with development of resistance to either first and second generations (linear and macrocyclic) PIs.

## Materials and Methods

### Study Population

This study included 1568 NS3-protease sequences (amino acids 1–181), either obtained in our laboratory (N = 32 from genotype 1a [N = 3], 1b [N = 3], 2a [N = 7], 3a [N = 16], 4 [N = 3]) or retrieved from GenBank, all isolated from PI-naive patients infected with HCV-genotypes 1a (N = 618), 1b (N = 471), 2 (N = 65, of which 14 HCV-2a, 40 HCV-2b, and 11 HCV-2c), 3 (N = 252, of which 249 HCV-3a and 3 HCV-3b), 4 (N = 51), 5 (N = 6), and 6 (N = 73).

To ensure the quality of the data, public-sequences were excluded from the analysis if: a) contained stop-codons in NS3-gene; b) contained ambiguities consisting of >2 bases per nucleotide position or >2 ambiguities per codon at individual drug-resistance associated position.

HCV genotype was assessed by phylogenetic analysis of NS3-sequences, aligning all retrieved sequences versus 15 reference-strains for the 6 different genotypes and most common subtypes included in the analysis (GenBank-accession-numbers: HCV-1a:NC_004102; HCV-1b:D90208; HCV-2a:AB047639/D00944; HCV-2b:D10988/AB030907; HCV-2c:D50409; HCV-2k:AB031663; HCV-3a:D17763/D28917; HCV-3b:D49374; HCV-3k:D63821; HCV-4:DQ418782; HCV-5:NC_009826; HCV-6:NC_009827). NS3-protease phylogenetic tree was estimated using the MEGA 5.0 package [40] by a Neighbour Joining approach, using the Kimura 2-parameter model, with a proportion of invariable sites and applying a gamma distribution.

### Protease Sequencing

Four home-made protocols for amplification and population sequencing of NS3-protease-gene for HCV-genotypes 1a-1b-2-3-4 were developed. The complete encoding NS3-region (181aa) was amplified from stored serum-samples by nested polymerase-chain-reaction (PCR) following a reverse-transcription reaction. HCV RNA was extracted using a standard commercial silica-gel membrane-binding method (QIAamp Viral-RNA Minikit; Qiagen, Valencia, CA), and the NS3-DNA-fragment was synthesized using the commercial SuperScript One-Step RT-PCR System (Invitrogen Corp, Carlsbad, CA). Primers used for amplification and sequencing are reported in Table S1. Two separate PCRs were performed for each sample. Reaction mixtures were prepared with 25 µl of RNA template, 8 µl of 5 mM Mg^2+^, 2 µl of DNase-RNase-free water, 0.75 µl of each primer at a concentration of 10 µM, 1 µl of RNase-out (40 U/ul), 1.5 µl of RT/Taq, 1 µl of dNTPs at a concentration of 10 mM for a total of 40 µl. Then, they were reverse-transcribed for 30 min at 45°C, denatured for 2 min at 94°C, and amplified by 40 cycles at 94°C for 30 seconds, 56°C for 30 seconds, and 68°C for 90 seconds. The amplified product was run on a 1% agarose-gel. When the product was not visible, a nested PCR was performed. Five µl of amplified product was denatured at 94°C for 3 min and amplified with 35 cycles at 94°C for 30 sec, 54°C for 30 sec, and 68°C for 45 sec, using the following reaction mix: 5 µl of 10 X Taq buffer, 4 µl of 25 mM Mg^2+^, 32.5 µl of DNase-RNase free water, 0.9 µl of each primer at a concentration of 10 µM, 1 µl of 10 mM dNTPs, 0.7 µl of Taq (5 U/µl) for a total of 45 µl. Positive samples were sequenced using the BigDye terminator v.3.1-cycle-sequencing-kit (Applied-Biosystems) and run on the automated-sequencer ABI-3100.

### Conservation Analysis

Analyzing 1568 sequences, NS3-protease HCV variability was firstly assessed by calculating the prevalence of the most common wild-type nucleotide at each position of NS3 gene. Afterwards, it was determined the impact of nucleotide variability on NS3-protein, evaluating the prevalence of wild-type and mutated amino acids.

Fourteen positions interested by resistance development to either linear or macrocyclic PIs were analyzed [8], [10], [20], [31]–[35], [41]. Resistance associated mutations (RAMs) have been divided into major and minor according to the FC *in vitro* >10 to at least one linear/macrocyclic PI. In particular, 19 major RAMs have been analyzed: 7 associated with both linear and macrocyclic PIs resistance (54A/S, 155K/Q/T, 156T/V), 5 exclusively related to linear compounds (36A/M, 55A, 170A/T), and 7 exclusively related to macrocyclic compounds (80K/R, 168A/H/T/V/E). Furthermore, 24 minor/secondary RAMs were analyzed 36L/G, 41R, 43S/V, 54V, 80L/H/G, 109K, 138T, 155I/M/S/G/L, 156G/S, 158I, 168I/G/N/Y and 175L.

### Structural Analysis

In order to visualize the distribution of conserved/variable NS3-residues, Protein Data Bank X-ray structures 3P8N and 2OC8 (available from http://www.rcsb.org/pdb) have been considered as 3D models of HCV-1b [42] and HCV-1a [43] NS3-protease respectively, and graphically inspected by PyMOL (The PyMol-Molecular-Graphics-System, ver.-1.3, Schrödinger, LLC). The crystallographic structures were selected considering the resolution of the models (3P8N 1,90 Å; 2OC8 2,66 Å) and excluding those crystals that showed a large number of deletions and mutations if compared to the reference sequences.

The evaluation of boceprevir-protease-interactions has been performed with Maestro-GUI (Maestro-Graphics-User-Interface, ver.-9.8, Schrödinger, LLC). To highlight the most relevant residues for the boceprevir targets recognition, the new computational approach GRID-Based-Pharmacophore-Model (GBPM) has been applied. Such a method, useful for designing pharmacophore models starting from detailed macromolecular structures, has been described in a recent publication [44]. In particular it was developed with the aim to generate pharmacophore models useful for QSAR and virtual screening experiments by means of an unbiased computational protocol. The GRID-based pharmacophore model is created in a 6-step procedure. The first one performs the PDB file pre-treatment producing three different model structures: the complex (subunits α+β), the receptor (subunit α) and the ligand (subunit β). The second step calculates the GRID molecular interaction fields (MIF) with a certain probe onto the three targets above reported. In the third step an energy comparison of the MIFs is performed by the GRID GRAB [45] utility, generating maps with focused information on the interaction areas. The fourth step is related to the identification of most relevant interaction points. With the aim to get a suitable model, these operations should be repeated using at least three different probes: a generic hydrophobic (DRY), an hydrogen bond acceptor (O) and an hydrogen bond donor (N1). In the fifth step the information obtained from the different probes are unified into a preliminary pharmacophore model. We carried out the GBPM analysis up to the fifth step of the procedure, in order to highlight the most involved residues in the recognition areas.

In the GRID [45] calculations the lone pairs, the tautomeric hydrogen atoms and torsion angles, relative to the sp3 oxygen atoms and the amide atoms, have been allowed to be settled on the basis of the probe influence, while the coordinates of all the other atoms have been considered rigid (directive MOVE = 0). Default values have been used for the other parameters.

In our analysis we used N1 (hydrogen-bond-donor), O (hydrogen-bond-acceptor) and DRY (hydrophobic) probes [44], [45].

The component interaction analysis was performed starting from the experimental HCV protease wild-type complex (PDB 2OC8) in the following conditions: a) OPLS2005 as force field; b) GB/SA water implicit solvation model; c) dielectric constant equal to 1; d) a binding pocket defined considering protease residues within 12Å from boceprevir (Maestro Graphics User Interface, ver. 9.8, Schrödinger, LLC).

Because the obtained global energy minimum GRID points (E_min_) were ranked in a wide range of values, graphical analysis of the GRID maps was carried out by considering, for each probe, an energy threshold (E_cut_) equal to 60% of the protease-boceprevir complex E_min_, as previously reported [46].

### Putative Secondary RNA Structure

A full length HCV-1b genome obtained by GenBank (accession number: AJ000009) was used for RNA secondary structures prediction by using the Mfold program at 37°C, available at the UNAFold server (http://mfold.rna.albany.edu) [47]. This algorithm, based on thermodynamics of RNA structures motifs, including base-paired intramolecular stems and unpaired loops, provides the identification of putative optimal minimum free energy structures. RNA structure models and free energy values were individually predicted using the original viral HCV-1b genome AJ000009, with and without the introduction in the NS3-protease coding region of specific resistance mutations at position 156: A156S, A156T, A156G, and A156V.

Secondary RNA structures were individually predicted by using also CONTRAfold-software, analyzing the NS3-fragment covering amino acid from 135 to 181. This software uses probabilistic parameters learned from a set of RNA secondary structures to predict base-pair probabilities and structures using the maximum expected accuracy approach [48], [49].

### Genetic Barrier Calculation

The genetic barrier for the evolution of any drug-resistance mutation was calculated according to a model previously described for HIV-1 and HBV [50], [51], considering not only the number of nucleotide substitutions, but also the nature of them (i.e. transitions *vs.* transversions). In summary, it was assigned a score of 1 to transitions (A↔G and C↔T) and a score of 2.5 to transversions (A↔C, A↔T, G↔C, G↔T), since transitions have been shown to occur, for steric reasons, on average 2.5 more frequently than transversions [50], [52]. An algorithm was built using Java-script to calculate the genetic barrier at each individual NS3 position. The pipeline used to estimate the genetic barrier for drug-resistance development have been described elsewhere [51]. Briefly, due to the degeneration of genetic code, each NS3 amino acid associated with drug-resistance can be encoded by more than one nucleotide codon. Therefore, starting from the *wild-type* codon detected in drug-naïve patients, we calculated a numerical score by summing the number of nucleotide transitions and/or transversions required to generate a specific RAM. As a result, we obtained different scores for each pathway of nucleotide substitutions required to generate a specific RAM. The minimal genetic barrier score for each drug resistance mutation analyzed was considered.

Only amino acid mutations with prevalence >1% and found in >2 patients were considered.

## Results

### NS3 Genetic Variability

**Figure 1:**
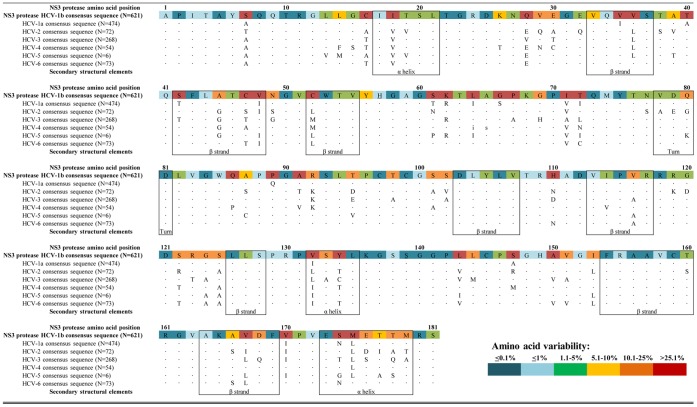
Amino acid sequence alignment of HCV genotypes 1–6 NS3 protease. The secondary structural elements identified from the HCV-1 NS3 crystal structures are reported at the bottom of the sequences. The consensus sequence of HCV-1b NS3-protease is shown as a reference, colored according to the frequency rate of mutations observed in 1568 HCV-sequences. HCV sequences reported are the consensus sequences obtained from the datasets included in the analysis. Secondary structural elements are indicated as reported in ViralZone (http://viralzone.expasy.org/). Catalytic residues (H57-D81-S139) are underlined. Identical amino acids among all HCV genotypes are indicated with dots “•”.

Overall, by analyzing 1568 NS3-protease sequences, the protease amino acidic sequence was moderately conserved, with 85/181 (47.0%) amino acids showing <1% amino acid variability among all HCV-genotypes, and 17.1% positions showing >25.1% variability (Fig. 1). Furthermore, amino-acid variability was almost absent (<0.1% variability) at 50/181 (27.6%) positions.

In the majority of cases, conserved amino acids clustered into small regions, comprising a number of 2 to 8 consecutive residues. For instance, we observed two highly conserved stretches encompassing NS3 positions 135–142 and 154–159 (Fig. 1). As expected, the catalytic-triad was highly conserved: residues D81 and S139 showed no amino acid variability, while the residue H57 was 100% conserved within HCV-1b strains (data not shown), and presented <1% variability among other HCV-genotypes. The catalytically oxyanion hole at G137 and the 4 residues involved in Zn^2+^ binding (C97-C99-C145-H149) were also highly conserved, showing <1% amino acid variability among all sequences analyzed (Fig. 1).

The comparison of consensus sequences between subtype 1b (used as reference in the present study) versus HCV-1a showed that 17 residues out of 181 were different among the HCV-1 subtypes, with some mutations found exclusively in HCV-1a and in none of the other HCV genotype (such as at positions 35-40-66-89) (Fig. 1).

Fourteen positions associated with resistance to either linear or macrocyclic PIs were considered [8], [10], [20], [31]–[35], [41]. In particular, 19 major RAMs were analyzed: 7 associated with both linear and macrocyclic PIs resistance (54A/S, 155K/Q/T, 156T/V), 5 exclusively related to linear compounds (36A/M, 55A, 170A/T), and 7 exclusively related to macrocyclic compounds (80K/R, 168A/H/T/V/E). Furthermore, 24 minor/secondary RAMs were analyzed 36L/G, 41R, 43S/V, 54V, 80L/H/G, 109K, 138T, 155I/M/S/G/L, 156G/S, 158I, 168I/G/N/Y and 175L.

Interestingly, 6 positions involved in major RAMs development showed a very high degree of both inter- and intra-genotype variability. Indeed, a different *wild-type* amino acid was detected among HCV-genotypes at positions 36, 80, 168 and 170, while positions 54 and 55 presented a noteworthy degree of intra-genotype variability. On the contrary, positions 41, 43, 109, 155, 156 and 158 had both inter- and intra-genotype amino acid variability <1%.

Notably, four RAMs associated with PIs treatment (80K/G, 36L and 175L) were found as natural polymorphisms in selected genotypes (Fig. 1). Indeed, the major RAM 80K (for macrocyclic compounds TMC435 and Asunaprevir) was detected in 41.6% of HCV-1a, in 100% of HCV-5 and in 20.6% of HCV-6 sequences, while the minor 80G was present in 94.4% HCV-2 sequences. Similarly, the minor RAM 36L was naturally present in 100% HCV-3-5 and >94% HCV-2-4, while the minor 175L RAM was present in 100% HCV-1a-3-5 and in >97% of HCV-2-4 sequences.

Also several positions associated with enhanced replication or compensatory effect if mutated (72-86-89-153-176) [53] were found to be highly variable. In particular, positions 72, 86, 89 and 176 had an amino acid variability >10%, with also evidences of differences in *wild-type* amino acids usage, while position 162 was highly conserved.

### Structural Insight of NS3 Protease

To better characterize the effect of HCV variability in the structure of NS3 protease and specifically in the binding-site to PIs, a NS3 protease-boceprevir contact analysis was carried out on an available HCV-1a NS3 protease-boceprevir complex model (PDB 2OC8). Several amino acidic residues, essential for boceprevir- and substrate-binding, were identified by structural and GRID-Based-Pharmacophore-Model (GBPM) approaches. In particular, the inhibitor was found to establish 3 hydrogen bonds with A157, single hydrogen bonds with residues Q41, G137, S139 and R155, and also numerous (>10) non-bonded contacts with several residues (H57-I132-L135-K136-G137-S139-F154-R155-A156-A157-V158) (Table 1 and Fig. S1). In addition, the protease residues H57, I132, S139, A156 and A157 were well identified at energy minimum threshold (data not shown), emphasizing their key role in enzyme catalytic activity and stabilization [54].

**Table 1:** Boceprevir interacting residues in the experimental HCV-1a NS3 protease-boceprevir complex model (PDB 2OC8).

NS3 residue^a^	Amino acid variability among HCV genotypes, (%)	Hydrogen bondswith boceprevir, N	Additional interactionswith boceprevir, N	Interaction type
**Q41**	0.5	1	2	El
**T42**	40.3		1	El
**F43**	0.1		4	vdW
**L44**	0.4		2	vdW
**V55**	1.2		1	vdW
**H57**	0.2		38	El, vdW
**R123**	16.3		8	vdW
**I132**	45.8		23	vdW
**L135**	0.1		17	vdW
**K136**	0.1		32	vdW
**G137**	0.1	1	12	El, vdW
**S138**	0.2		4	El, vdW
**S139**	0.1	1	31	El, vdW
**F154**	0.1		18	vdW
**R155**	0.9	1	33	El, vdW
**A156**	0.1		57	El, vdW
**A157**	0.1	3	47	El, vdW
**V158**	0.4		15	vdW
**C159**	0.1		5	vdW
**D168**	16.8		6	vdW

a The residues are reported as in the wild-type HCV-1a sequence.

El, electrostatic non-bonded contacts; vdW, van der Waals non-bonded contacts.

**Figure 2:**
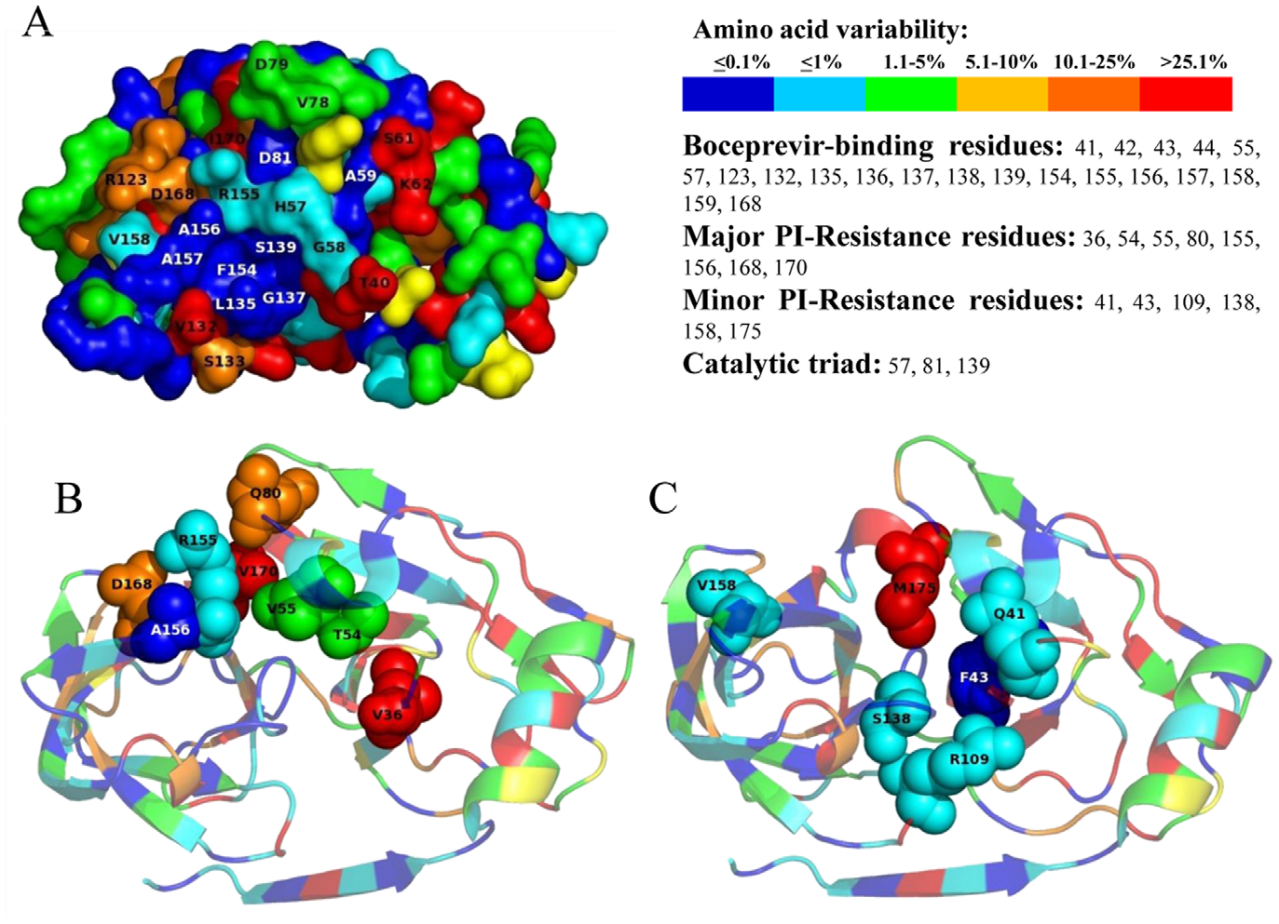
Conservation of HCV NS3 protease from PI-naïve HCV infected patients. Panel (**A**) reports the molecular surface structure of HCV-1b NS3-protease, colored according to the frequency rate of mutations observed in all 1568 HCV-sequences. The catalytic-triad and residues located inside and in proximity of the hydrophobic-core of the NS3-protease are reported. Panels (**B**) and (**C**) show a co-crystalized boceprevir-HCV-1a protease structure, colored according to the amino acid conservation observed in all HCV-sequences.

Interestingly, among all identified NS3 residues essential for boceprevir-binding by structural and GBPM-analysis, the majority (Q41-F43-L44-H57-L135-K136-G137-S138-S139-F154-R155-A156-A157-V158-C159) were found highly conserved among all HCV-genotypes (amino acid variability <1%; Table 1, Fig. 1 and Fig. 2). Differently, residues at positions 42, 123, 132 and 168 were highly polymorphic (amino acid variability = 40.3%, 16.3%, 45.8% and 16.8%, respectively) (Table 1, Fig. 1). Interestingly, at these positions, HCV-3 sequences presented different *wild-type* amino acids in respect to HCV-1b sequences, and at position 123 and 168 this resulted in a non-conservative change of charge (Fig. 1). Indeed, HCV-3 showed a polar Threonine (T) instead of a positively-charged Arginine (R) as *wild-type* amino acid at position 123, and a non-charged Glutamine (Q) instead of a negatively-charged Aspartic acid (D) as *wild-type* amino acid at position 168. Analyzing the tertiary HCV-1b NS3-protease structure, residues 123 and 168 were found adjacent to each other, in direct proximity with R155 and A156 residues, two of the most important for protease-drug interaction and resistance development to linear and macrocyclic PIs (Fig. 2, panel A). Differently, mutations at position 168 (A/E/G/H/T/Y) have been associated with high-moderate level of resistance to all macrocyclic PIs of first generation.

All together, these structural analyses highlighted the presence of some genotype-specific polymorphisms at positions close to the NS3-protease catalytic site, but also underlined the existence of many highly conserved residues involved in the catalytic functionality of the enzyme, and thus excellent target for a focused pharmacophoric design.

### NS3 Genetic Variability and RNA Secondary-structure

**Figure 3:**
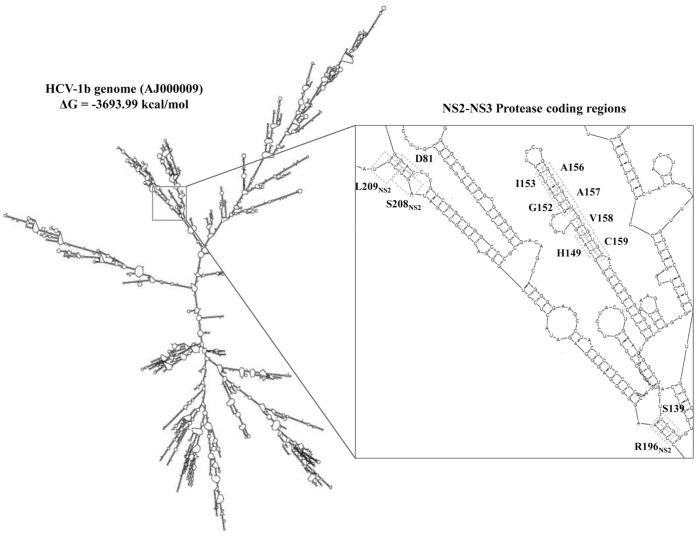
Predicted RNA secondary structure of HCV NS3 serine protease sequence. The RNA secondary structure of HCV-1b genome was predicted by the Mfold program. The viral genome sequence modelled, was an isolate phylogenetically classified as HCV 1b (accession number: AJ000009). A very stable stem-loop, including codons for amino acid positions 145 to 163 (corresponding at NS3 Protease coding region), was reported into box.

By using CONTRAfold-software, a first analysis of all nucleotide-sequences of the NS3-protease coding-region showed the formation of a very complex RNA secondary-structure, almost exclusively organized in highly stable paired-stems. Analyzing the structure in more detail, by using the Mfold-software and one entire HCV1-b genome sequence, we noticed that base-paired RNA stretches were often composed by highly conserved codon pairs in the protease coding region (Fig. 3). For instance, the highly conserved codons for catalytic residues D81 and S139 were base-paired with the conserved S208-L209, e R196 NS2-residues, respectively (Fig. 3). Differently, the codon for the catalytic residue H57, which presented a high synonymous nucleotide variability (>50%), in our RNA model was only partially base-paired, facing the highly variable codon for P67 (data not shown).

**Figure 4:**
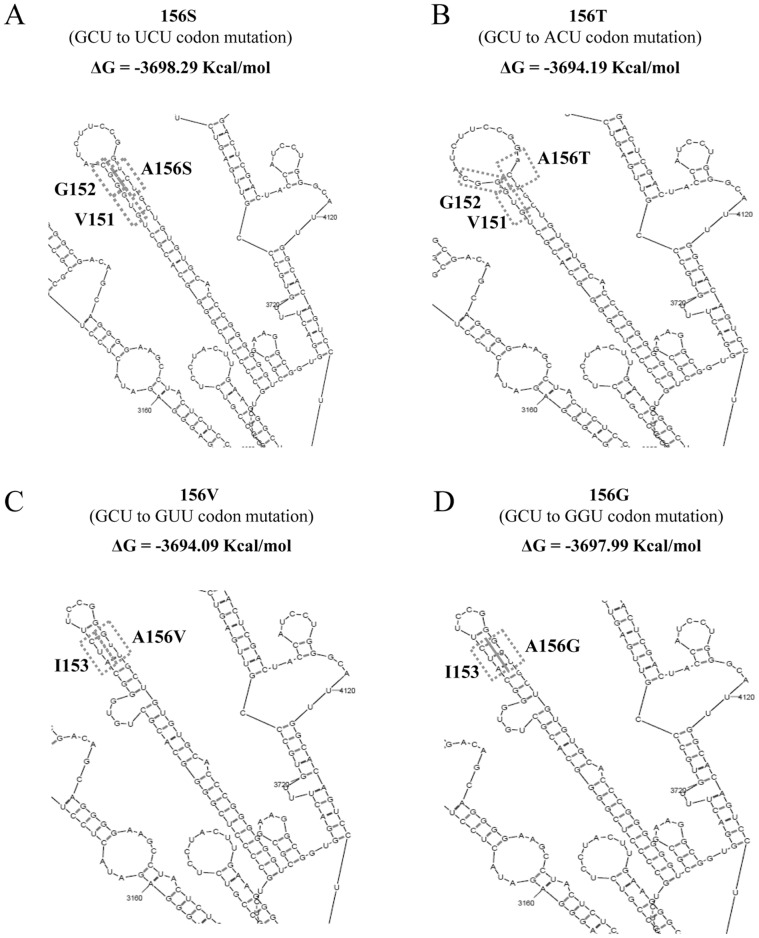
Predicted RNA secondary structure of HCV NS3 serine protease mutated at position 156. The RNA structures and the relative free energy values were individually predicted by using Mfold software. The NS3-fragment extrapolated from viral genome modelled was considered, specifically analyzing the resistance mutations at position 156: A156S (**A**), A156T (**B**), A156V (**C**) and A156G (**D**). Codons for amino acid at position 156 and for those base-paired with position 156 are reported into panels.

Within the NS3 coding region, a very stable stem-loop, including codons for amino acid positions 145 to 163, was observed (Fig. 3). Highly conserved codons (amino acid variability ≤1%) were base-paired within this loop, while codons with higher variability among HCV-genotypes, such as those for amino acids at positions 150-151-155, were not base-paired. Interestingly, the codon for residue A156 (*gcu*), associated to resistance to all linear and some macrocyclic PIs (including the second generation MK-5172), was found to be base-paired with the highly conserved codon for I153 (*auc*). When the RAMs 156S (*ucu* codon), 156T (*acu* codon), 156V (*guu* codon), or 156G (*ggu* codon) were introduced in the NS3-protease coding region, our model of RNA stem-loop conformation was not perturbed (Fig. 4). Indeed, all simulation models were associated with similar delta values of free-energy (ΔG) decrease. The A156S development was associated with the higher decrease of ΔG values, from 3693.99 kcal/mol of the *wild-type* model to -3698.29 kcal/mol of the A156S harboring model, suddenly followed by A156G (ΔG = −3697.99 kcal/mol), A156T (ΔG = −3694.19 kcal/mol), and A156V (ΔG = −3694.09 kcal/mol). This small decrease of ΔG values indicates a persistence of structural-stability and also suggests that mutations 156S/T/V/G, if occurring during virological failure to PIs, should not drastically alter the HCV RNA secondary structure. These structural and entropic results were confirmed using CONTRAfold software (data not shown).

### Genetic Barrier for PIs Resistance

The genetic barrier for the development of RAMs was explored on the whole data set of 1568 NS3-protease sequences. Starting from each *wild-type* codon detected in the dataset of sequences obtained from PI-naïve patients, we calculated a numerical score by summing the number of nucleotide transitions and/or transversions required to generate a specific RAM. As a result, we obtained different scores for each pathway of nucleotide substitutions required to generate a specific RAM. The minimal genetic barrier score for each drug resistance mutation analyzed was considered.

**Figure 5:**
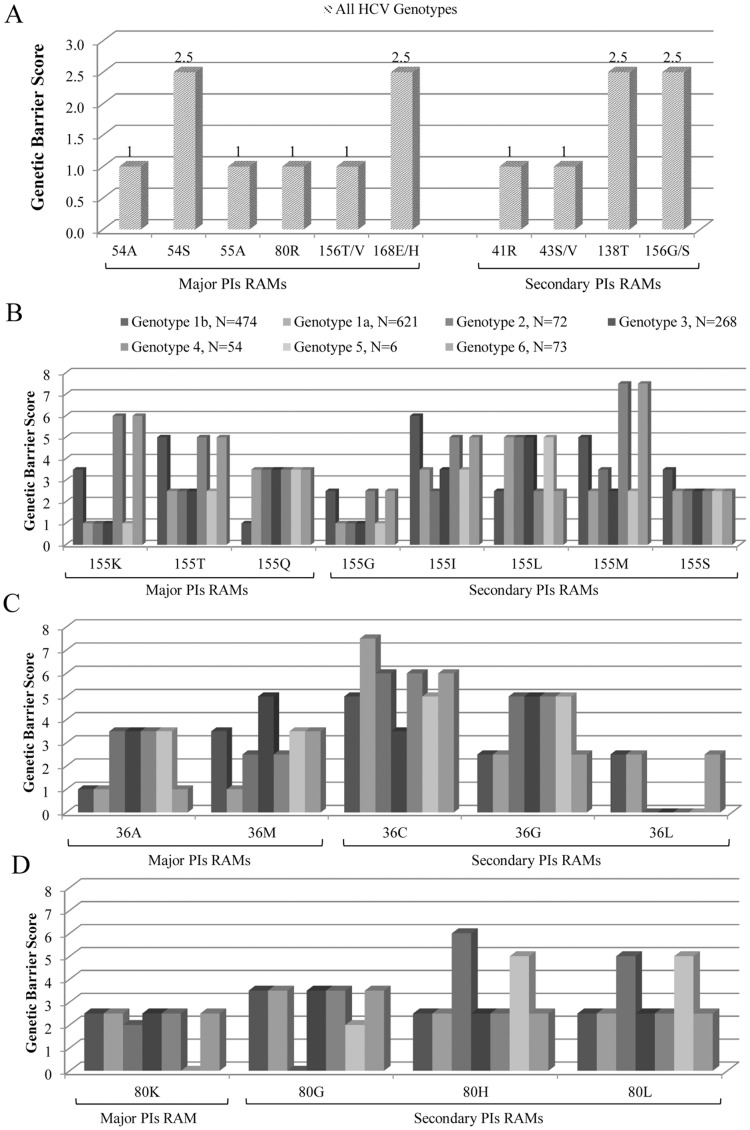
Calculated genetic-barrier for resistance-mutations. Mutations reported in panel (**A**) are those for which the calculated genetic-barrier was not affected by inter-genotype variability. Histograms in panel below represent the calculated genetic-barrier score for RAMs at positions 155 (**B**), 36 (**C**), and 80 (**D**). The score was calculated by summing the number of transitions (score = 1) and transversions (score = 2.5) required for the generation of any degenerated codon associated with drug-resistance, starting from the predominant wild-type codon found in each HCV-genotype.

Regardless of HCV genotype, major RAMs 55A (for boceprevir), 54A/S (for boceprevir or telaprevir), 80R (for TMC435 or asunaprevir), 156T/V (for all linear and several macrocyclic PIs, including MK5172 second generation) and 168E/H (for all first generation macrocyclic PIs) needed only one nucleotide substitution (in the majority of cases, a transition, score = 1) to be generated and were thus associated with the lowest values of genetic barrier (Fig. 5 panel A). Accordingly, this may justify their very rapid selection under PI-treatment. Also several secondary RAMs had a low genetic barrier to development in all HCV genotypes, requiring only 1 transition (such as danoprevir mutations 41R and 43S/V: score = 1) or 1 transversion (138T and 156G/S: score = 2.5).

Differently, either nucleotide and/or amino acid variability among HCV-genotypes affected the calculated genetic barrier for the development of other major and minor RAMs (such as at positions 36, 80, 109, 155, 168, 170, 175). A detailed analysis of codon variability for major and minor RAMs is reported in Table 2 and in Table 3, respectively.

**Table 2:** Codon variability at HCV NS3 positions associated with major drug resistance to PIs and its impact on the genetic barrier to drug resistance development in HCV-genotypes 1–6.

Position	WT Amino^a^	WT Codon	Proportion of *wild-type* and polymorphic codons in HCV-genotype, N(%) b							Resistance mutations					
			1a (N = 621)	1b (N = 474)	2 (N = 72)	3 (N = 268)	4 (N = 54)	5 (N = 6)	6 (N = 73)	Mutation	Codon^c^	Minimal score^d^	Mutation	Codon^c^	Minimal score^d^
***Major mutations associated with linear and macrocyclic PIs resistance***															
54	T	ACT	**506 (81.5)**	**372 (78.5)**	19 (26.4)	**259 (96.6)**	14 (25.9)	0 (0.0)	**25 (34.2)**	**T54A**	GCT	1	**T54S**	AGT/TCT	2.5
54		ACA	3 (0.5)	4 (0.8)	**33 (45.8)**	0 (0.0)	0 (0.0)	**6 (100)**	17 (23.3)	**T54A**	GCA	1	**T54S**	TCA	2.5
54		ACC	102 (16.4)	91 (19.2)	11 (15.3)	9 (3.4)	**27 (50)**	0 (0.0)	14 (19.2)	**T54A**	GCC	1	**T54S**	AGC/TCC	2.5
54		ACG	0 (0.0)	3 (0.6)	11 (15.3)	0 (0.0)	8 (14.8)	0 (0.0)	17 (23.3)	**T54A**	GCG	1	**T54S**	TCG	2.5
155	R	CGG	1 (0.2)	**414 (87.3)**	27 (37.5)	0 (0.0)	1 (1.9)	0 (0.0)	1 (1.4)	**R155K**	AAG	3.5	**R155T**	ACG	5
155		AGA	110 (17.7)	0 (0.0)	**37 (51.4)**	28 (10.4)	0 (0.0)	0 (0.0)	0 (0.0)	**R155K**	AAA	1	**R155T**	ACA	2.5
155		AGG	**503 (81.0)**	19 (4.0)	3 (4.2)	**239 (89.2)**	0 (0.0)	**6 (100)**	1 (1.4)	**R155K**	AAG	1	**R155T**	ACG	2.5
155		CGA	0 (0.0)	38 (8.0)	3 (4.2)	0 (0.0)	12 (22.2)	0 (0.0)	24 (32.9)	**R155K**	AAA	3.5	**R155T**	ACA	5
155		CGC	0 (0.0)	1 (0.2)	1 (1.4)	0 (0.0)	**23 (42.6)**	0 (0.0)	**31 (42.5)**	**R155K**	AAA/G	6	**R155T**	ACC	5
155		CGT	0 (0.0)	0 (0.0)	1 (1.4)	0 (0.0)	16 (29.6)	0 (0.0)	17 (23.3)	**R155K**	AAA/G	6	**R155T**	ACT	5
155	R	CGG	1 (0.2)	**414 (87.3)**	27 (37.5)	0 (0.0)	1 (1.9)	0 (0.0)	1 (1.4)	**R155Q**	CAG	1			
155		AGA	110 (17.7)	0 (0.0)	**37 (51.4)**	28 (10.4)	0 (0.0)	0 (0.0)	0 (0.0)	**R155Q**	CAA	3.5			
155		AGG	**503 (81.0)**	19 (4.0)	3 (4.2)	**239 (89.2)**	0 (0.0)	**6 (100)**	1 (1.4)	**R155Q**	CAG	3.5			
155		CGA	0 (0.0)	38 (8.0)	3 (4.2)	0 (0.0)	12 (22.2)	0 (0.0)	24 (32.9)	**R155Q**	CAA	1			
155		CGC	0 (0.0)	1 (0.2)	1 (1.4)	0 (0.0)	**23 (42.6)**	0 (0.0)	**31 (42.5)**	**R155Q**	CAA	3.5			
155		CGT	0 (0.0)	0 (0.0)	1 (1.4)	0 (0.0)	16 (29.6)	0 (0.0)	17 (23.3)	**R155Q**	CAG	3.5			
156	A	GCT	28 (4.5)	**386 (81.4)**	3 (4.2)	**211 (78.7)**	7 (13)	**4 (66.7)**	**22 (30.1)**	**A156T**	ACA/GTT	1	**A156V**	ACA/GTT	1
156		GCA	1 (0.2)	0 (0.0)	28 (38.9)	2 (0.7)	3 (5.6)	0 (0.0)	15 (20.5)	**A156T**	ACC/GTA	1	**A156V**	ACC/GTA	1
156		GCC	**591 (95.2)**	87 (18.4)	0 (0.0)	52 (19.4)	11 (20.4)	2 (33.3)	14 (19.2)	**A156T**	ACG/GTC	1	**A156V**	ACG/GTC	1
156		GCG	1 (0.2)	1 (0.2)	**41 (56.9)**	2 (0.7)	**30 (55.6)**	0 (0.0)	21 (28.8)	**A156T**	ACT/GTG	1	**A156V**	ACT/GTG	1
***Major mutations associated with linear PIs resistance***															
36	V	GTT	0 (0.0)	**278 (58.6)**	5 (6.9)	0 (0.0)	0 (0.0)	0 (0.0)	10 (13.7)	**V36A**	GCT	1	**V36M**	ATG	3.5
36		GTA	28 (4.5)	3 (0.6)	0 (0.0)	0 (0.0)	0 (0.0)	0 (0.0)	6 (8.2)	**V36A**	GCA	1	**V36M**	ATG	2
36		GTC	1 (0.2)	178 (37.6)	0 (0.0)	0 (0.0)	0 (0.0)	0 (0.0)	**26 (35.6)**	**V36A**	GCC	1	**V36M**	ATG	3.5
36		GTG	**584 (93.9)**	6 (1.3)	0 (0.0)	0 (0.0)	0 (0.0)	0 (0.0)	14 (19.2)	**V36A**	GCG	1	**V36M**	ATG	1
36	L	CTA	0 (0.0)	0 (0.0)	2 (2.8)	1 (0.4)	4 (7.4)	1 (16.7)	4 (5.5)	**L36A**	GCA	3.5	**L36M**	ATG	3.5
36		CTC	0 (0.0)	4 (0.8)	0 (0.0)	12 (4.5)	0 (0.0)	0 (0.0)	7 (9.6)	**L36A**	GCC	3.5	**L36M**	ATG	5
36		CTG	3 (0.5)	2 (0.4)	**49 (68.1)**	1 (0.4)	**36 (66.7)**	2 (33.3)	1 (1.4)	**L36A**	GCG	3.5	**L36M**	ATG	2.5
36		CTT	0 (0.0)	1 (0.2)	0 (0.0)	**253 (94.4)**	1 (1.9)	0 (0.0)	0 (0.0)	**L36A**	GCT	3.5	**L36M**	ATG	5
36		TTA	0 (0.0)	0 (0.0)	0 (0.0)	0 (0.0)	2 (3.7)	**3 (50.0)**	0 (0.0)	**L36A**	GCA	3.5	**L36M**	ATG	3.5
36		TTG	3 (0.5)	1 (0.2)	17 (23.6)	1 (0.4)	9 (16.7)	0 (0.0)	1 (1.4)	**L36A**	GCG	3.5	**L36M**	ATG	2.5
36	I	ATC	0 (0.0)	1 (0.2)	0 (0.0)	0 (0.0)	0 (0.0)	0 (0.0)	2 (2.7)	**I36A**	GCC	2	**I36M**	ATG	2.5
55	V	GTC	**579 (93.2)**	**423 (89.2)**	20 (27.8)	14 (5.2)	**23 (42.6)**	**3 (50.0)**	31 (42.5)	**V55A**	GCA	1			
55		GTA	0 (0.0)	2 (0.4)	**33 (45.8)**	1 (0.4)	6 (11.1)	0 (0.0)	5 (6.8)	**V55A**	GCG	1			
55		GTG	1 (0.2)	6 (1.3)	7 (9.7)	0 (0.0)	16 (29.6)	0 (0.0)	9 (12.3)	**V55A**	GCT	1			
55		GTT	24 (3.9)	43 (9.1)	12 (16.7)	**252 (94.0)**	7 (13.0)	1 (16.7)	**40 (54.8)**	**V55A**	GCC	1			
55	A	GCT	2 (0.3)	0 (0.0)	0 (0.0)	0 (0.0)	0 (0.0)	0 (0.0)	0 (0.0)	**-**	**-**	**-**			
55		GCC	8 (1.3)	0 (0.0)	0 (0.0)	0 (0.0)	0 (0.0)	0 (0.0)	0 (0.0)	**-**	**-**	**-**			
55	I	ATC	7 (1.1)	0 (0.0)	0 (0.0)	0 (0.0)	0 (0.0)	0 (0.0)	0 (0.0)	**I55A**	GCC	2			
55	L	CTC	0 (0.0)	0 (0.0)	0 (0.0)	0 (0.0)	0 (0.0)	2 (33.3)	0 (0.0)	**L55A**	GCA	3.5			
170	V	GTG	0 (0.0)	**163 (34.4)**	1 (1.4)	1 (0.4)	**21 (38.9)**	0 (0.0)	**33 (45.2)**	**V170A**	GCG	1	**V170T**	ACG	2
170		GTA	0 (0.0)	148 (31.2)	4 (5.6)	26 (9.7)	6 (11.1)	0 (0.0)	3 (4.1)	**V170A**	GCA	1	**V170T**	ACA	2
170		GTC	14 (2.3)	3 (0.6)	6 (8.3)	0 (0.0)	19 (35.2)	**2 (33.3)**	9 (12.3)	**V170A**	GCC	1	**V170T**	ACC	2
170		GTT	1 (0.2)	19 (4)	0 (0.0)	0 (0.0)	3 (5.6)	0 (0.0)	6 (8.2)	**V170A**	GCT	1	**V170T**	ACT	2
170	I	ATA	1 (0.2)	138 (29.1)	0 (0.0)	**240 (89.6)**	0 (0.0)	0 (0.0)	4 (5.5)	**I170A**	GCA	2	**I170T**	ACA	1
170		ATC	**588 (94.7)**	3 (0.6)	**64 (88.9)**	0 (0.0)	1 (1.9)	**2 (33.3)**	16 (21.9)	**I170A**	GCC	2	**I170T**	ACC	1
170		ATT	17 (2.7)	0 (0.0)	1 (1.4)	1 (0.4)	1 (1.9)	**2 (33.3)**	1 (1.4)	**I170A**	GCT	2	**I170T**	ACT	1
***Major mutations associated with macrocyclic PIs resistance***															
80	Q	CAG	56 (9.0)	**391 (82.5)**	5 (6.9)	**185 (69.0)**	5 (9.3)	0 (0.0)	**30 (41.1)**	**Q80K**	AAG	2.5	**Q80R**	CGG	1
80		CAA	**300 (48.3)**	61 (12.9)	0 (0.0)	83 (31.0)	**46 (85.2)**	0 (0.0)	27 (37.0)	**Q80K**	AAA	2.5	**Q80R**	CGA	1
80	G	GGA	1 (0.2)	0 (0.0)	23 (31.9)	0 (0.0)	0 (0.0)	0 (0.0)	0 (0.0)	**G80K**	AAA	2	**G80R**	AGA	1
80		GGC	0 (0.0)	0 (0.0)	4 (5.6)	0 (0.0)	0 (0.0)	0 (0.0)	0 (0.0)	**G80K**	AAA/AAG	6	**G80R**	GCG	5
80		GGG	0 (0.0)	3 (0.6)	**41 (56.9)**	0 (0.0)	0 (0.0)	0 (0.0)	0 (0.0)	**G80K**	AAG	2	**G80R**	AGG	1
80	K	AAA	186 (30)	0 (0.0)	0 (0.0)	0 (0.0)	0 (0.0)	**6 (100)**	1 (1.4)	**-**	**-**		**K80R**	AGA	1
80		AAG	72 (11.6)	1 (0.2)	0 (0.0)	0 (0.0)	0 (0.0)	0 (0.0)	14 (19.2)	**-**	**-**		**K80R**	AGG	1
168	D	GAC	**590 (95.0)**	**426 (89.9)**	**55 (76.4)**	0 (0.0)	**47 (87)**	0 (0.0)	**41 (56.2)**	**D168A**	GCC	2.5	**D168H**	CAC	2.5
168		GAT	29 (4.7)	45 (9.5)	17 (23.6)	0 (0.0)	5 (9.3)	**4 (66.7)**	30 (41.1)	**D168A**	GCT	2.5	**D168H**	CAT	2.5
168	E	GAA	2 (0.3)	3 (0.6)	0 (0.0)	0 (0.0)	0 (0.0)	0 (0.0)	0 (0.0)	**E168A**	GCA	2.5	**E168H**	CAT/CAC	5
168		GAG	0 (0.0)	0 (0.0)	0 (0.0)	0 (0.0)	0 (0.0)	2 (33.3)	2 (2.7)	**E168A**	GCG	2.5	**E168H**	CAT/CAC	5
168	Q	CAG	0 (0.0)	0 (0.0)	0 (0.0)	**214 (79.9)**	0 (0.0)	0 (0.0)	0 (0.0)	**Q168A**	GCG	5	**Q168H**	CAA/G	2.5
168		CAA	0 (0.0)	0 (0.0)	0 (0.0)	4 (1.5)	0 (0.0)	0 (0.0)	0 (0.0)	**Q168A**	GCA	5	**Q168H**	CAA/G	2.5
168	R	CGG	0 (0.0)	0 (0.0)	0 (0.0)	1 (0.4)	0 (0.0)	0 (0.0)	0 (0.0)	**R168A**	GCG	5	**R168H**	CAC/T	3.5
168	D	GAC	**590 (95)**	**426 (89.9)**	**55 (76.4)**	0 (0.0)	**47 (87.0)**	0 (0.0)	**41 (56.2)**	**D168T**	ACC	3.5	**D168V**	GTC	2.5
168		GAT	29 (4.7)	45 (9.5)	17 (23.6)	0 (0.0)	5(9.3)	**4 (66.7)**	30 (41.1)	**D168T**	ACT	3.5	**D168V**	GTT	2.5
168	E	GAA	2 (0.3)	3 (0.6)	0 (0.0)	0 (0.0)	0 (0.0)	0 (0.0)	0 (0.0)	**E168T**	ACA	3.5	**E168V**	GTA	2.5
168		GAG	0 (0.0)	0 (0.0)	0 (0.0)	0 (0.0)	0 (0.0)	2 (33.3)	2 (2.7)	**E168T**	ACG	3.5	**E168V**	GTC	2.5
168	Q	CAG	0 (0.0)	0 (0.0)	0 (0.0)	**214 (79.9)**	0 (0.0)	0 (0.0)	0 (0.0)	**Q168T**	ACG	5	**Q168V**	GTG	5
168		CAA	0 (0.0)	0 (0.0)	0 (0.0)	4 (1.5)	0 (0.0)	0 (0.0)	0 (0.0)	**Q168T**	ACA	5	**Q168V**	GTA	5
168	R	CGG	0 (0.0)	0 (0.0)	0 (0.0)	1 (0.4)	0 (0.0)	0 (0.0)	0 (0.0)	**R168T**	ACG	5	**R168V**	GTG	5
168	D	GAC	**590 (95.0)**	**426 (89.9)**	**55 (76.4)**	0 (0.0)	**47 (87.0)**	0 (0.0)	**41 (56.2)**	**D168E**	GAA/G	2.5			
168		GAT	29 (4.7)	45 (9.5)	17 (23.6)	0 (0.0)	5 (9.3)	**4 (66.7)**	30 (41.1)	**D168E**	GAA/G	2.5			
168	E	GAA	2(0.3)	3(0.6)	0 (0.0)	0 (0.0)	0 (0.0)	0 (0.0)	0 (0.0)	**-**	-	-			
168		GAG	0 (0.0)	0 (0.0)	0 (0.0)	0 (0.0)	0 (0.0)	2 (33.3)	3 (2.19)	**-**	-	-			
168	Q	CAG	0 (0.0)	0 (0.0)	0 (0.0)	**214 (79.9)**	0 (0.0)	0 (0.0)	0 (0.0)	**Q168E**	GAG	2.5			
168		CAA	0 (0.0)	0 (0.0)	0 (0.0)	4 (1.5)	0 (0.0)	0 (0.0)	0 (0.0)	**Q168E**	GAA	2.5			
168	R	CGG	0 (0.0)	0 (0.0)	0 (0.0)	1 (0.4)	0 (0.0)	0 (0.0)	0 (0.0)	**R168E**	GAG	3.5			

a The wild-type amino-acid of HCV genotype 1b at each position associated with drug resistance is shown.

b Predominant wild-type codon for each genotype is reported in bold.

c Codon for drug-resistance mutation requiring the lowest number of transitions/transversions starting from the wild-type or polymorphic codon detected in drug-naïve patients.

d Minimal numerical score obtained by summing the number of nucleotide transitions and/or transversions (scored as 1 and 2.5, respectively, see methods) required to generate the specific drug-resistance mutation.

WT, wild-type.

**Table 3:** Codon variability at HCV NS3 positions associated with minor drug resistance to PIs and its impact on the genetic barrier to drug resistance development in HCV-genotypes 1–6.

Position		WT Amino^a^		WT Codon		Proportion of *wild-type* and polymorphic codons in HCV-genotype, N(%) b											Resistance mutations										
						1a (N = 621)	1b (N = 474)	2 (N = 72)		3 (N = 268)	4 (N = 54)		5 (N = 6)		6 (N = 73)		Mutation		Codon^c^		Minimal score^d^		Mutation		Codon^c^	Minimal score^d^	
***Minor mutations associated with linear and macrocyclic PIs resistance***																											
41		Q		CAA		**367 (59.1)**	**412 (86.9)**	**43 (59.7)**		6 (2.2)	2 (3.7)	**6 (100)**			32 (43.8)		**Q41R**		CGA		1						
41				CAG		248 (39.9)	60 (12.7)	29 (40.3)		**262 (97.8)**	**49 (90.7)**	0 (0.0)			**41 (56.2)**		**Q41R**		CGG		1						
41		H		CAC		3 (0.5)	1 (0.2)	0 (0.0)		0 (0.0)	0 (0.0)	0 (0.0)			0 (0.0)		**H41R**		CGC		1						
41				CAT		3 (0.5)	0 (0.0)	0 (0.0)		0 (0.0)	0 (0.0)	0 (0.0)			0 (0.0)		**H41R**		CGT		1						
43		F		TTC		**562 (90.5)**	**462 (97.5)**	**63 (87.5)**		**267 (99.6)**	**40 (74.1)**	**3 (50)**			**68 (93.2)**		**F43S**		TCC		1		**F43V**		TCC	1	
43				TTT		59 (9.5)	12 (2.5)	9 (12.5)		0 (0.0)	11 (20.4)	**3 (50)**			5 (6.8)		**F43S**		TCT		1		**F43V**		TCT	1	
155		R		CGG		1 (0.2)	**414 (87.3)**	27 (37.5)		0 (0.0)	1 (1.9)	0 (0.0)			1 (1.4)		**R155I**		ATA		6		**R155G**		GGG	2.5	
155				AGA		110 (17.7)	0 (0.0)	**37 (51.4)**		28 (10.4)	0 (0.0)	0 (0.0)			0 (0.0)		**R155I**		ATA		2.5		**R155G**		GGA	1	
155				AGG		**503 (81.0)**	19 (4.0)	3 (4.2)		**239 (89.2)**	0 (0.0)	**6 (100)**			1 (1.4)		**R155I**		ATA		3.5		**R155G**		GGG	1	
155				CGA		0 (0.0)	38 (8.0)	3 (4.2)		0 (0.0)	12 (22.2)	0 (0.0)			24 (32.9)		**R155I**		ATA		5		**R155G**		GGA	2.5	
155				CGC		0 (0.0)	1 (0.2)	1 (1.4)		0 (0.0)	**23 (42.6)**	0 (0.0)			**31 (42.5)**		**R155I**		ATC		5		**R155G**		GGC	2.5	
155				CGT		0 (0.0)	0 (0.0)	1 (1.4)		0 (0.0)	16 (29.6)	0 (0.0)			17 (23.3)		**R155I**		ATT		5		**R155G**		GGT	2.5	
155		R		CGG		1 (0.2)	**414 (87.3)**	27 (37.5)		0 (0.0)	1 (1.9)	0 (0.0)			1 (1.4)		**R155M**		ATG		5		**R155L**		CTG	2.5	
155				AGA		110 (17.7)	0 (0.0)	**37 (51.4)**		28 (10.4)	0 (0.0)	0 (0.0)			0 (0.0)		**R155M**		ATG		3.5		**R155L**		T/CTA	5	
155				AGG		**503 (81.0)**	19 (4.0)	3 (4.2)		**239 (89.2)**	0 (0.0)	**6 (100)**			1 (1.4)		**R155M**		ATG		2.5		**R155L**		C/TTG	5	
155				CGA		0 (0.0)	38 (8.0)	3 (4.2)		0 (0.0)	12 (22.2)	0 (0.0)			24 (32.9)		**R155M**		ATG		6		**R155L**		CTA	2.5	
155				CGC		0 (0.0)	1 (0.2)	1 (1.4)		0 (0.0)	**23 (42.6)**	0 (0.0)			**31 (42.5)**		**R155M**		ATG		7.5		**R155L**		CTC	2.5	
155				CGT		0 (0.0)	0 (0.0)	1 (1.4)		0 (0.0)	16 (29.6)	0 (0.0)			17 (23.3)		**R155M**		ATG		7.5		**R155L**		CTT	2.5	
155		R		CGG		1 (0.2)	**414 (87.3)**	27 (37.5)		0 (0.0)	1 (1.9)	0 (0.0)			1 (1.4)		**R155S**		TCG		3.5						
155				AGA		110 (17.7)	0 (0.0)	**37 (51.4)**		28 (10.4)	0 (0.0)	0 (0.0)			0 (0.0)		**R155S**		AGC/T		2.5						
155				AGG		**503 (81.0)**	19 (4.0)	3 (4.2)		**239 (89.2)**	0 (0.0)	**6 (100)**			1 (1.4)		**R155S**		AGC/T		2.5						
155				CGA		0 (0.0)	38 (8.0)	3 (4.2)		0 (0.0)	12 (22.2)	0 (0.0)			24 (32.9)		**R155S**		TCA		3.5						
155				CGC		0 (0.0)	1 (0.2)	1 (1.4)		0 (0.0)	**23 (42.6)**	0 (0.0)			**31 (42.5)**		**R155S**		AGC		2.5						
155				CGT		0 (0.0)	0 (0.0)	1 (1.4)		0 (0.0)	16 (29.6)	0 (0.0)			17 (23.3)		**R155S**		AGT		2.5						
156		A		GCT		28 (4.5)	**386 (81.4)**	3 (4.2)		**211 (78.7)**	7 (13)	**4 (66.7)**			**22 (30.1)**		**A156S**		TCT		2.5		**A156G**		GGT	2.5	
156				GCA		1 (0.2)	0 (0.0)	28 (38.9)		2 (0.7)	3 (5.6)	0 (0.0)			15 (20.5)		**A156S**		TCA		2.5		**A156G**		GGA	2.5	
156				GCC		**591 (95.2)**	87 (18.4)	0 (0.0)		52 (19.4)	11 (20.4)	2 (33.3)			14 (19.2)		**A156S**		TCC		2.5		**A156G**		GGC	2.5	
156				GCG		1 (0.2)	1 (0.2)	**41 (56.9)**		2 (0.7)	**30 (55.6)**	0 (0.0)			21 (28.8)		**A156S**		TCG		2.5		**A156G**		GGG	2.5	
***Minor mutations associated with linear PIs resistance***																											
36		V	GTT			0 (0.0)	**278 (58.6)**	5 (6.9)		0 (0.0)	0 (0.0)	0 (0.0)			10 (13.7)		**V36G**		GGT		2.5		**V36L**		CTT	2.5	
36			GTA			28 (4.5)	3 (0.6)	0 (0.0)		0 (0.0)	0 (0.0)	0 (0.0)			6 (8.2)		**V36G**		GGA		2.5		**V36L**		C/TTA	2.5	
36			GTC			1 (0.2)	178 (37.6)	0 (0.0)		0 (0.0)	0 (0.0)	0 (0.0)			**26 (35.6)**		**V36G**		GGC		2.5		**V36L**		CTC	2.5	
36			GTG			**584 (93.9)**	6 (1.3)	0 (0.0)		0 (0.0)	0 (0.0)	0 (0.0)			14 (19.2)		**V36G**		GGG		2.5		**V36L**		C/TTG	2.5	
36		L	CTA			0 (0.0)	0 (0.0)	2 (2.8)		1 (0.4)	4 (7.4)	1 (16.7)			4 (5.5)		**L36G**		GGA		5		**-**		-	-	
36			CTC			0 (0.0)	4 (0.8)	0 (0.0)		12 (4.5)	0 (0.0)	0 (0.0)			7 (9.6)		**L36G**		GGC		5		**-**		-	-	
36			CTG			3 (0.5)	2 (0.4)	**49 (68.1)**		1 (0.4)	**36 (66.7)**	2 (33.3)			1 (1.4)		**L36G**		GGG		5		**-**		-	-	
36			CTT			0 (0.0)	1 (0.2)	0 (0.0)		**253 (94.4)**	1 (1.9)	0 (0.0)			0 (0.0)		**L36G**		GGT		5		**-**		-	-	
36			TTA			0 (0.0)	0 (0.0)	0 (0.0)		0 (0.0)	2 (3.7)	**3 (50.0)**			0 (0.0)		**L36G**		GGA		5		**-**		-	-	
36			TTG			3 (0.5)	1 (0.2)	17 (23.6)		1 (0.4)	9 (16.7)	0 (0.0)			1 (1.4)		**L36G**		GGG		5		**-**		-	-	
36		I	ATC			0 (0.0)	1 (0.2)	0 (0.0)		0 (0.0)	0 (0.0)	0 (0.0)			2 (2.7)		**I36G**		GGC		3.5		**I36L**		CTC	2.5	
54		T	ACT			**506 (81.5)**	**372 (78.5)**	19 (26.4)		**259 (96.6)**	14 (25.9)	0 (0.0)			**25 (34.2)**		**T54V**		GTT		2						
54			ACA			3 (0.5)	4 (0.8)	**33 (45.8)**		0 (0.0)	0 (0.0)	**6 (100)**			17 (23.3)		**T54V**		GTA		2						
54			ACC			102 (16.4)	91 (19.2)	11 (15.3)		9 (3.4)	**27 (50)**	0 (0.0)			14 (19.2)		**T54V**		GTC		2						
54			ACG			0 (0.0)	3 (0.6)	11 (15.3)		0 (0.0)	8 (14.8)	0 (0.0)			17 (23.3)		**T54V**		GTG		2						
109		R	AGG			**612 (98.6)**	**341 (71.9)**	2 (2.8)		0 (0.0)	**38 (70.4)**	**4 (66.7)**			**46 (63.0)**		**R109K**		AAG		1						
109			AGA			7 (1.1)	127 (26.8)	1 (1.4)		0 (0.0)	10 (18.5)	0 (0.0)			10 (13.7)		**R109K**		AAA		1						
109			CGA			0 (0.0)	0 (0.0)	**44 (61.1)**		3 (1.1)	1 (1.9)	0 (0.0)			2 (2.7)		**R109K**		AAA		3.5						
109			CGC			0 (0.0)	0 (0.0)	2 (2.8)		**243 (90.7)**	1 (1.9)	0 (0.0)			4 (5.5)		**R109K**		AAA/G		6						
109			CGG			1 (0.2)	4 (0.8)	25 (34.7)		0 (0.0)	4 (7.4)	2 (33.3)			11 (15.1)		**R109K**		AAG		3.5						
109			CGT			0 (0.0)	0 (0.0)	0 (0.0)		24 (9.0)	0 (0.0)	0 (0.0)			0 (0.0)		**R109K**		AAA/G		6						
158		V	GTG			**569 (91.6)**	**399 (84.2)**	**69 (95.8)**		**254 (94.8)**	**49 (90.7)**	**6 (100)**			**28 (38.4)**		**V158I**		ATA		2						
158			GTA			52 (8.4)	72 (15.2)	0 (0.0)		12 (4.5)	3 (5.6)	0 (0.0)			23 (31.5)		**V158I**		ATA		1						
158			GTC			0 (0.0)	2 (0.4)	1 (1.4)		1 (0.4)	0 (0.0)	0 (0.0)			16 (21.9)		**V158I**		ATC		1						
158			GTT			0 (0.0)	1 (0.2)	0 (0.0)		0 (0.0)	0 (0.0)	0 (0.0)			6 (8.2)		**V158I**		ATT		1						
175		M	ATG			0 (0.0)	**472 (99.6)**	2 (2.8)		0 (0.0)	1 (1.9)	0 (0.0)			**73 (100)**		**M175L**		C/TTG		2.5						
175		L	CTA			**548 (88.2)**	0 (0.0)	0 (0.0)		1 (0.4)	1 (1.9)	0 (0.0)			0 (0.0)		–		–		–						
175			CTC			0 (0.0)	0 (0.0)	**53 (73.6)**		35 (13.1)	7 (13)	0 (0.0)			0 (0.0)		–		–		–						
175			CTG			44 (7.1)	1 (0.2)	1 (1.4)		3 (1.1)	1 (1.9)	**4 (66.7)**			0 (0.0)		–		–		–						
175			CTT			2 (0.3)	0 (0.0)	14 (19.4)		**230 (85.8)**	**39 (72.2)**	0 (0.0)			0 (0.0)		–		–		–						
175			TTA			23 (3.7)	0 (0.0)	0 (0.0)		0 (0.0)	2 (3.7)	0 (0.0)			0 (0.0)		–		–		–						
175			TTG			8 (1.3)	1 (0.2)	0 (0.0)		1 (0.4)	0 (0.0)	2 (33.3)			0 (0.0)		–		–		–						
175		I	ATC			0 (0.0)	0 (0.0)	2 (2.8)		0 (0.0)	0 (0.0)	0 (0.0)			0 (0.0)		I175L		CTC		2.5						
***Minor mutations associated with macrocyclic PIs resistance***																											
80	Q		CAG		56 (9.0)		**391 (82.5)**	5(6.9)	**185(69.0)**		5 (9.3)	0 (0.0)		**30 (41.1)**		**Q80L**		CTG		2.5		**Q80H**		CAC/CAT			2.5
80			CAA		**300 (48.3)**		61 (12.9)	0 (0.0)	83 (31.0)		**46 (85.2)**	0 (0.0)		27 (37.0)		**Q80L**		CTA		2.5		**Q80H**		CAC/CAT			2.5
80	G		GGG		0 (0.0)		3 (0.6)	**41(56.9)**	0 (0.0)		0 (0.0)	0 (0.0)		0 (0.0)		**G80L**		CTG/TTG		5		**G80H**		CAC/CAT			6
80			GGA		1 (0.2)		0 (0.0)	23(31.9)	0 (0.0)		0 (0.0)	0 (0.0)		0 (0.0)		**G80L**		CTA/TTA		5		**G80H**		CAC/CAT			6
80			GGC		0 (0.0)		0 (0.0)	4 (5.6)	0 (0.0)		0 (0.0)	0 (0.0)		0 (0.0)		**G80L**		CTC		3.5		**G80H**		CAC			5
80	K		AAA		186 (30.0)		0 (0.0)	0 (0.0)	0 (0.0)		0 (0.0)	**6 (100)**		1 (1.4)		**K80L**		CTA/TTA		5		**K80H**		CAC/CAT			5
80			AAG		72 (11.6)		1 (0.2)	0 (0.0)	0 (0.0)		0 (0.0)	0 (0.0)		14 (19.2)		**K80L**		CTG/TTG		5		**K80H**		CAC/CAT			5
80	Q		CAG		56 (9.0)		**391 (82.5)**	5(6.9)	**185(69.0)**		5 (9.3)	0 (0.0)		**30 (41.1)**		**Q80G**		GGG		3.5							
80			CAA		**300 (48.3)**		61 (12.9)	0 (0.0)	83 (31.0)		**46 (85.2)**	0 (0.0)		27 (37.0)		**Q80G**		GGA		3.5							
80	G		GGG		0 (0.0)		3 (0.6)	**41(56.9)**	0 (0.0)		0 (0.0)	0 (0.0)		0 (0.0)		–		–		–							
80			GGA		1 (0.2)		0 (0.0)	23(31.9)	0 (0.0)		0 (0.0)	0 (0.0)		0 (0.0)		–		–		–							
80			GGC		0 (0.0)		0 (0.0)	4 (5.6)	0 (0.0)		0 (0.0)	0 (0.0)		0 (0.0)		–		–		–							
80	K		AAA		186 (30.0)		0 (0.0)	0 (0.0)	0 (0.0)		0 (0.0)	**6 (100)**		1 (1.4)		K80G		GGA		2							
80			AAG		72 (11.6)		1 (0.2)	0 (0.0)	0 (0.0)		0 (0.0)	0 (0.0)		14 (19.2)		**K80G**		GGG		2							
138	S		TCT		34 (5.5)		**351 (74.1)**	5 (6.9)	7 (2.6)		**27 (50.0)**	0 (0.0)		4 (5.5)		**S138T**		ACT		2.5							
138	S		TCA		4 (0.6)		3 (0.6)	**39 (54.2)**	1 (0.4)		7 (13.0)	**3 (37.5)**		17 (23.3)		**S138T**		ACA		2.5							
138	S		TCC		**583 (93.9)**		117 (24.7)	19 (26.4)	**260 (97.0)**		17 (31.5)	**3 (37.5)**		**29 (39.7)**		**S138T**		ACC		2.5							
138	S		TCG		0 (0.0)		2 (0.4)	9 (12.5)	0 (0.0)		2 (3.7)	2 (25.0)		22 (30.1)		**S138T**		ACG		2.5							
168	D		GAC		**590 (95.0)**		**426 (89.9)**	**55 (76.4)**	0 (0.0)		**47 (87.0)**	0 (0.0)		**41 (56.2)**		**D168I**		ATC		3.5		**D168G**		GGT			2
168			GAT		29 (4.7)		45 (9.5)	17 (23.6)	0 (0.0)		5 (9.3)	**4 (66.7)**		30 (41.1)		**D168I**		ATT		3.5		**D168G**		GGT			1
168	E		GAG		0 (0.0)		0 (0.0)	0 (0.0)	0 (0.0)		0 (0.0)	2(33.3)		3 (2.19)		**E168I**		ATA		4.5		**E168G**		GGG			1
168	Q		CAG		0 (0.0)		0 (0.0)	0 (0.0)	**214 (79.9)**		0 (0.0)	0 (0.0)		0 (0.0)		**Q168I**		ATA		6		**Q168G**		GGA			3.5
168			CAA		0 (0.0)		0 (0.0)	0 (0.0)	4 (1.5)		0 (0.0)	0 (0.0)		0 (0.0)		**Q168I**		ATA		5		**Q168G**		GGG			3.5
168	R		CGG		0 (0.0)		0 (0.0)	0 (0.0)	1 (0.4)		0 (0.0)	0 (0.0)		0 (0.0)		**R168I**		ATA		6		**R168G**		GGG			2.5
168	D		GAC		**590 (95.0)**		**426 (89.9)**	**55 (76.4)**	0 (0.0)		**47 (87.0)**	0 (0.0)		**41 (56.2)**		**D168N**		AAC		1		**D168Y**		TAC			2.5
168			GAT		29 (4.7)		45 (9.5)	17 (23.6)	0 (0.0)		5 (9.3)	**4 (66.7)**		30 (41.1)		**D168N**		AAT		1		**D168Y**		TAT			2.5
168	E		GAG		0 (0.0)		0 (0.0)	0 (0.0)	0 (0.0)		0 (0.0)	2(33.3)		3 (2.19)		**E168N**		AAC/T		3.5		**E168Y**		TAC/T			5
168	Q		CAG		0 (0.0)		0 (0.0)	0 (0.0)	**214 (79.9)**		0 (0.0)	0 (0.0)		0 (0.0)		**Q168N**		AAC/T		5		**Q168Y**		TAC/T			3.5
168			CAA		0 (0.0)		0 (0.0)	0 (0.0)	4 (1.5)		0 (0.0)	0 (0.0)		0 (0.0)		**Q168N**		AAC/T		5		**Q168Y**		TAC/T			3.5
168	R		CGG		0 (0.0)		0 (0.0)	0 (0.0)	1 (0.4)		0 (0.0)	0 (0.0)		0 (0.0)		**R168N**		AAC/T		6		**R168Y**		TAC/T			4.5

a The wild-type amino-acid of HCV genotype 1b at each position associated with drug resistance is shown.

b Predominant wild-type codon for each genotype is reported in bold.

c Codon for drug-resistance mutation requiring the lowest number of transitions/transversions starting from the wild-type or polymorphic codon detected in drug-naïve patients.

d Minimal numerical score obtained by summing the number of nucleotide transitions and/or transversions (scored as 1 and 2.5, respectively, see methods) required to generate the specific drug-resistance mutation.

WT, wild-type.

For instance, the NS3-residue R155, critical for both linear and macrocyclic PIs resistance, showed high degree of nucleotide variability (Table 2), leading to a different genetic barrier for the development of all RAMs at this position (Fig. 5 panel B). Notably, the calculated genetic barrier for the major R155T mutation, associated with high-level of resistance to linear PIs, was found to be lower in HCV-1a-2-3-5 genotypes (score = 2.5), in comparison to HCV-1b-4-6 (score = 5). Similarly, the development of R155K mutation, associated with high resistance to linear and macrocyclic PIs, required only 1 transition for its potential development in HCV-1a-2-3-5 genotypes (score = 1), in comparison to 1 transversion or more substitutions required in HCV-1b-4-6 genotypes (scores = 2.5-3.5-6). Also some minor RAMs (R155G/I/M) showed a lower genetic barrier for their development in HCV-1a-2-3-4-5-6 genotypes in comparison to HCV-1b. Differently, the potential development of the major RAM R155Q, associated with resistance to linear PIs and danoprevir, required only one transition in HCV-1b (score = 1) in comparison to other genotypes, such as HCV-1a-2-3-5, where 2 substitutions were required (scores = 3.5) (Table 2 and Fig. 5, panel B).

According to the different *wild-type* codon usage at position 36, the calculated genetic barrier for the development of RAMs at this position varied among HCV-genotypes (Table 2, Fig. 5 panel C). For instance, the potential development of 36 M (known to compensate impaired viral fitness of R155K mutation) had a reduced genetic barrier in HCV-1a (score = 1), but also in HCV-2-4 (score = 2.5), in comparison to HCV-1b (score = 3.5), HCV-3 (score = 5), and HCV-5-6 (score = 3.5).

A large nucleotide and amino acid variability among HCV-genotypes was also found at position 170, where HCV-1a-2-3-5 showed an Isoleucine (I) as a predominant *wild-type* amino acid instead of the Valine (V) predominantly found in HCV-1b-4-6. As a consequence, HCV-1a-2-3-5 could preferably develop the major boceprevir RAM 170T (with genetic barrier score = 1), while HCV-1b-4-6 could favor the development of 170A (also in this case, score = 1) (Table 2).

Also the two positions associated with major resistance to macrocyclic PI (80 and 168) were highly variable among genotypes (Fig. 1 and Fig. 5, panel D). In particular, at position 80, genotypes HCV-1a-1b-3-4-6 showed a Q as *wild-type* amino acid, and consequently the development of major Q80K and Q80R RAMs will potentially require just a nucleotide substitution (score = 2.5 and 1, respectively). On the contrary, HCV-2 harbored the minor RAM 80G as *wild-type,* and presented a calculated genetic barrier score of 1 for the development of 80R and of 2 for the development of 80K (two transitions). Lastly HCV-5 already had the major 80K as *wild-type*, and had a genetic barrier for major 80R development scored as 1.

As already mentioned before, near all HCV-3 sequences analyzed showed Q168 as *wild-type* amino acid, instead of D168 (Fig. 1 and Table 2). As a consequence, HCV-3 showed an increased genetic barrier for the major variant 168V (score = 5 for HCV-3 *versus* 2.5 for all other HCV-genotypes), 168T (score = 5 *versus* 3.5), and 168A (score = 5 *versus* 2.5) and the minor variant 168I (score = 6 *versus* 3.5) and 168Y (score = 3.5 *versus* 2.5), 168N (score = 5 *versus* 1).

Taken together, these results indicate that the high level of variability in codon usage among HCV-genotypes can favor genotype-specific pathways for resistance-mutations development. This can result in different responsiveness of HCV-genotypes to PIs and very rapid selection for specific resistance patterns for both linear and macrocyclic PIs.

### Discussion

Analyzing more than 1500 HCV NS3-protease sequences, a high degree of genetic variability among all HCV-genotypes was found in PI-naïve HCV-infected patients, with only 85/181 (47.0%) conserved amino acids. This genetic heterogeneity among genotypes translated into significant molecular and structural differences, making HCV-genotypes, and even subtypes, differently sensitive to PIs treatment and differently prone to the development of PI resistance-mutations, for both linear and macrocyclic compounds. Indeed, the linear PI telaprevir showed less efficacy against HCV-2, and almost no efficacy against HCV-3-4-5 genotypes *in vitro* and *in vivo* [10], [24]–[26], and similar results were also obtained for macrocyclic inhibitors, such as danoprevir, vaniprevir and TMC435 [10], [24], [26].

As a first consequence of HCV sequence heterogeneity, we observed that four resistance-mutations (80K/G and 36L-175L) were already present, as natural polymorphisms, in selected genotypes. In particular, the major RAM 80K (for macrocyclic compounds TMC435 and Asunaprevir) was detected in 41.6% of HCV-1a, in 100% of HCV-5 and in 20.6% of HCV-6 sequences. Secondly, a different codon usage among genotypes led to a different genetic-barrier for the development of some major and minor RAMs at positions 36-80-109-155-168-170.

Notably, among all HCV-genotypes, the more difficult-to-treat HCV-3 presented several polymorphisms at positions close to the PI-binding site (42-45-123-132-133-134-168-170), which probably might be related to the low antiviral efficacy of several PIs observed *in vivo* and *in vitro* against this genotype [10], [24], [26], [33]. In particular, different wild-type amino acids at positions 123 and 168 resulted in non-conservative changes of charge. In co-crystalized structures of PIs and HCV-1 NS3-protease, the negatively charged D168 forms strong salt bridges with positively-charged residues R123 and R155 [20]. It has been proposed that mutations at either positions 155 or 168 could disrupt this salt bridge and affect the interaction with PIs, potentially leading to drug-resistance [20]. The substitution of D168 residue in HCV-3 with the polar uncharged Q168, and the replacement of R123 with the polar T123 can thus abrogate these key structural salt bridges, potentially altering the active site conformation of NS3 protease, and in turn impact the HCV-3 sensitivity to PIs.

Furthermore, HCV-3, together with HCV-2-4-5 genotypes, also presented two minor RAMs as natural polymorphisms (36L and 175L), known to confer low-level resistance to boceprevir and/or telaprevir *in vitro* [23], [25]. Interestingly, both residues 36 and 175 are located near the protease catalytic domain of HCV NS3, but not close to the boceprevir and telaprevir binding sites in their respective complexes with HCV NS3-NS4 protease (Fig. 2) [20]. Probably, even if mutations at position 36 and 175 should not be directly involved in resistance to PIs, they can influence the viral replication capacity. For instance, viruses with mutations V36A/L/M (as well as with other PI-resistance mutations such as R109K and D168E) demonstrated a comparable fitness to wild type reference virus [55]. However, since no crystallized structures are to date available for non-1 HCV proteases, the overall impact of such polymorphisms on the three-dimensional protein structure (and functionality) will need further investigations.

It is important to mention that very recent data demonstrated a pan-genotypic activity of the second generation macrocyclic PI MK-5172, even against HCV-3 genotype (Barnard R, presented at International Congress of Viral Hepatitis 2012, abstract n° 79340). Furthermore, MK-5172 retained activity also against HCV-1 viral strains harbouring key first generation PI RAMs, thus providing a great opportunity for patients infected with all different HCV-genotypes, including those without virological response to previous regimens.

Beside HCV-3, also other genotypes showed remarkable sequence differences from HCV-1b. Of particular interest were those genotype-specific amino acid variations affecting residues associated to macrocyclic and linear PIs-resistance (i.e. 36-54-55-80-168-170-175) or located in proximity of the PI-binding pocket (40-42-45-122-123-132-133-134).

For instance, HCV-1a and HCV-1b consensus sequences showed different wild-type amino acids at 17/181 (9.4%) NS3-protease positions, including some (i.e. 72-80-89-175) associated with resistance, enhanced replication or compensatory effects if mutated [35], [53]. This amino acidic variability (together with the nucleotide one) may potentially facilitate viral breakthrough and selection of specific resistant variants, that have been indeed observed consistently more frequently in patients infected with HCV-1a than HCV-1b, using both linear and macrocyclic PIs [27], [29].

On the other hand, according to our GBPM structural analysis, highly conserved NS3-protease positions among all HCV genotypes were those pivotal for enzyme functionality and stability, such as the catalytic-triad (H57-D81-S139), the oxyanion hole at G137 and the residues involved in Zn^2+^ binding (C97-C99-C145-H149), and also comprised the majority of residues essential for boceprevir-binding (Q41-F43-L44-H57-L135-K136-G137-S138-S139-F154-R155-A156-A157-V158-C159) [36], [42]. Interestingly, we also observed two highly conserved stretches encompassing NS3 positions 135–142 and 154–159 that could assist in the rational design of new HCV inhibitors with more favourable resistance profiles.

A correlation among conserved NS3 amino acid residues and base-paired organization on the putative RNA secondary structure was also observed. Indeed, highly conserved positions at both amino acid and nucleotide levels were located in highly stable RNA paired stems. Probably, the requirement for base-pairing in these structures severely limits the number of “neutral” sites in the genome, constraining neutral HCV drift, since even synonymous mutations could potentially affect and disrupt the RNA-folding [56].

Interestingly, in our predicted RNA structure model, the conserved codon for resistance-associated residue A156 [10] was base-paired with the conserved codon for residue I153. The presence of RAMs at this position (156S/T/G/V), associated to resistance to all linear and some macrocyclic PIs (including the second generation MK-5172) [29], [33], [57], did not perturb the overall RNA structural conformation and was associated with a delta free-energy decrease similar to that observed in the *wild-type* model, suggesting that the selection of such RAMs might determine a phenotypic drug-resistance without altering the secondary RNA-structure stability.

Another specific aim of the study was to compare the genetic barrier for the evolution of PIs resistance among all HCV genotypes. The calculation of the genetic barrier was performed considering not only the number of nucleotide substitutions, but also the nature of them (i.e. transitions, score = 1 vs. transversions, score = 2.5), according to recently published papers [50]–[52].

Analyzing all HCV sequences, the calculated genetic-barrier for the potential development of some RAMs to both linear and macrocyclic PIs was found to be lower in HCV-1a (36M-155G/I/K/M/S/T-170T), HCV-2 (36M-80K-155G/I/K/S/T-170T), HCV-3 (155G/I/K/M/S/T-170T), HCV-4-6 (155I/S/L), and HCV-5 (80G-155G/I/K/M/S/T), in comparison to HCV-1b genotype.

Differently, regardless of HCV-genotype, 15/37 RAMs analyzed were associated with very low genetic-barrier scores, requiring only one transition or transversion for their development (41R- 43S-54A-55A-156V/T-168N: score = 1; while 43C-54S-156S/G-168Y/E/H/A: score = 2.5).

All together, these results help explaining experimental and clinical observations, indicating that mutations appearing rapidly and frequently in PI-treated patients are actually those with a lower genetic barrier in the specific genotype/subtype considered. Indeed, in both telaprevir and/or boceprevir failing patients, the most common resistance mutations detected in HCV-1a infected patients were V36M, T54S, and R155K (all score = 1), whereas mutations T54A/S, V55A, A156S, and V170A (all score = 1 or 2.5) were specifically developed in HCV-1b patients [27], [29], [39] (Barnard R. et al., presented at AASLD 2011).

Furthermore, classically the genetic barrier calculation is performed referring to the most prevalent wild-type codon found in each genotype. Nevertheless, as it appears clearly from Table 2 and Table 3, the variability of codon usage exists at high level even within the single genotypes. For instance, we found 41.6% of HCV-1a sequences harboring the RAM 80K, and 4% of HCV-1b sequences with a reduced genetic barrier (score = 1) to develop R155K, suggesting that also individual isolates may differently respond to treatment and develop specific PI resistance mutations. At this regard, it is important to mention that natural HCV resistance has been described in few reports [53], [58]–[61], with a rare (<1%) natural presence of 155K found by population sequencing, exclusively in patients infected with HCV-1a [53], [58]–[61].

In conclusion, the high degree of HCV genetic variability makes HCV-genotypes, and even subtypes, differently prone to responsiveness to PIs and to the development of linear and macrocyclic RAMs. Learning also from the anti-HIV treatment experiences, the HCV genotypic resistant test will thus provide to clinicians important information for the management of HCV infection and for the individual tailoring of antiretroviral therapy. In this direction, a better knowledge of the extend of genetic variability among genotypes could assist the identification of RAMs with higher probability of development in that particular setting, highlighting patients with a higher risk of failure.

## Supporting Information

Figure S1 **2D representation of boceprevir interactions in the HCV-1 NS3-protease binding pocket within 5Å (PDB 2OC8).** Hydrogen bonds are reported as light magenta lines. Grey, green, cyan, pink and violet areas are related, respectively, to non polar uncharged, hydrophobic, polar uncharged, polar negatively charged and polar positively charged protease residues.
(DOC)

Table S1 **Primers for amplification and sequencing of NS3 protease of HCV-genotypes 1-2-3-4.**
(DOC)
