# Supplementary figures and images for: HCV Genotypes Are Differently Prone to the Development of Resistance to Linear and Macrocyclic Protease Inhibitors
Source: PLoS One. 2012 Jul 6;7(7):e39652. doi: 10.1371/journal.pone.0039652 (PMC3391197; doi:10.1371/journal.pone.0039652)

**
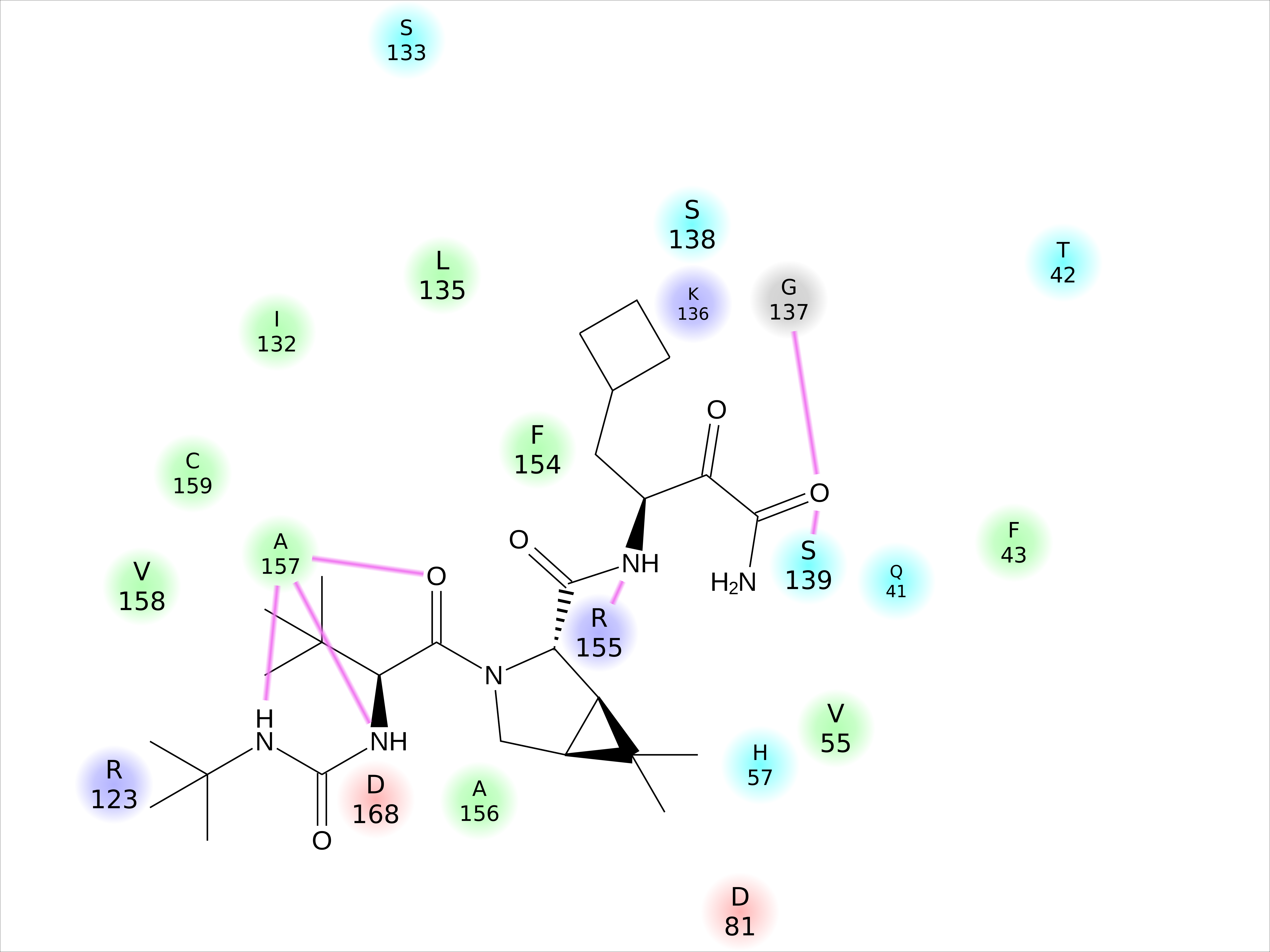
**

Supplement: Figure S1 — 2D representation of boceprevir interactions in the HCV-1 NS3-protease binding pocket within 5Å (PDB 2OC8). Hydrogen bonds are reported as light magenta lines. Grey, green, cyan, pink and violet areas are related, respectively, to non polar uncharged, hydrophobic, polar uncharged, polar negatively charged and polar positively charged protease residues. (DOC) [file pone.0039652.s001.doc]
