# Supplementary material for: HCV Genotypes Are Differently Prone to the Development of Resistance to Linear and Macrocyclic Protease Inhibitors
Source: PLoS One. 2012 Jul 6;7(7):e39652. doi: 10.1371/journal.pone.0039652 (PMC3391197; doi:10.1371/journal.pone.0039652)
Supplement: Table S1 — Primers for amplification and sequencing of NS3 protease of HCV-genotypes 1-2-3-4. (DOC) [file pone.0039652.s002.doc]

**Table S1.** Primers for amplification and sequencing of NS3 protease of HCV-genotypes 1-2-3-4

| **Primers** | **HCV Genotype** | | | |
| --- | --- | --- | --- | --- |
|  | **HCV-1** | **HCV-2** | **HCV-3** | **HCV-4** |
| **For1** | GTGCCSTACTTYGTGCGCG | GYCCGATGGAGARGAARGTCATCG | GCGGAGATATYCTTTGCGGGC | GGCAATGARATCYTGCTCGGSC |
| **Rev1** | CCGTCGGCAAGGAACTTGCC | GGCCCAGCTGATGGMTAYACC | TGCTAGTRGCGTCTTGGGCATG | GGTCYARGACCGTGCCTATGCC |
| **For2** | GGAGACYAAGCTCATYACSTGGGG | CCTGRTARGTCTGGGGCACAG | GGCCTTCTTGGGACTATTGTGACC | GGGGTGGAGRCTSCTTGCC |
| **Rev2** | CMGGSACCTTGGTGCTCTT | ACTGTYCCGATGCCRAGRATGGT | CGGTCGGGGCATGAAGRTACC | GCTCTTGCCACTTCCYGTTGG |
| **Seq** | ACCAATGTAGACCARGACCTCG | GACAACAGCACRCCACCRGC | CTTTTGCYACACCTCTGGTGCACA | TCCACCGCTTTAGCYACCCC |
